# The Effect of Finishing and Polishing Sequences on The Surface Roughness of Three Different Nanocomposites and Composite/Enamel and Composite/Cementum Interfaces

**DOI:** 10.3390/nano10071339

**Published:** 2020-07-09

**Authors:** Ksenia Babina, Maria Polyakova, Inna Sokhova, Vladlena Doroshina, Marianna Arakelyan, Nina Novozhilova

**Affiliations:** Department of Therapeutic Dentistry, I.M. Sechenov First Moscow State Medical University (Sechenov University), 119991 Moscow, Russia; polyakova_m_a_1@staff.sechenov.ru (M.P.); sokhova_i_a@staff.sechenov.ru (I.S.); doroshina_v_yu@staff.sechenov.ru (V.D.); arakelyan_m_g@staff.sechenov.ru (M.A.); novozhilova_n_e@staff.sechenov.ru (N.N.)

**Keywords:** surface roughness, nanocomposite resins, dental finishing and polishing, composite/enamel interface, composite/cementum interface, contact stylus profilometry

## Abstract

The purpose of this study was to investigate the effect of final surface treatment and dental composite type on the roughness of the composite surface, composite/enamel interface, and composite/cementum interface, as well as on the polishing time. Class V cavities prepared in extracted teeth (*n* = 126) were restored using one of the three nanohybrid composites with different filler sizes. The specimens were randomly assigned to three different finishing and polishing sequences. The roughness (Ra) of the investigated surfaces was measured using the contact profilometer. The time required to achieve visible gloss was documented. The data were analyzed using ANOVA with Tukey’s post hoc test (*p* < 0.05). There was no significant influence of the composite type on the restoration surface roughness (*p* = 0.088), while the polishing method had a significant impact (*p* < 0.001). The Ra of the composites ranged between 0.08 µm and 0.29 µm, with the lowest values (0.09 µm ± 0.05 µm) found in the aluminum oxide disc group (*p* < 0.001). The time to achieve a visible composite gloss was influenced by the polishing method, composite type, and interactions between these factors (*p* < 0.001). The interface roughness was significantly greater than that of the composite surface (*p* < 0.001), and depended on the composite type and polishing system employed.

## 1. Introduction

Nanotechnology applications in dentistry have shown constantly increasing interest, with a high number of studies on the topic. Nanocomposites are the most commonly used restorative materials in dentistry, due to their excellent mechanical characteristics [1], stable physicochemical assessment potential [2], high flexural properties [3], and potential remineralizing capabilities [4]. Depending on their chemistry and filler type, these materials exhibit a variety of handling properties and physical characteristics suitable for different clinical situations [5]. Based on the size of the filler particles, nanocomposites can be classified into nanofilled and nanohybrid resins. Nanofills contain uniform nanometric particles, whereas nanohybrids include various particles ranging in size from the micrometer to nanometer scale [6].

The esthetic results of these materials are strongly influenced by the final surface treatment [5]. An accurate finishing and polishing procedure is also crucial to enhance the longevity of composite restorations [7,8]. Finishing allows one to create the proper anatomy and occlusal morphology of the restoration, as well as a tight tooth-composite interface; polishing allows the elimination of scratches and a reduction in surface roughness [6]. Smooth surfaces reduce plaque accumulation, gingival irritation, recurrent caries, and the discoloration of restored teeth over the long term [7,9,10]. Poor surface quality, on the contrary, can enhance bacterial adhesion, decrease the wear resistance of the restoration [10,11], and cause irritation of the tongue, lips, and cheeks [12]. Another crucial factor for achieving predictable results is marginal finishing [13] since plaque accumulation around the restoration margins may lead to restoration failure [14].

Studies have shown that the effects of finishing and polishing procedures depend directly on both the restorative material and the polishing system [6,15]. The composite’s polishability is influenced by the resin matrix, as well as by filler size, shape, and loading. Relatively soft resin wears faster than harder filler particles, which prevent homogenous abrasion and result in surface irregularities. Large-sized filler particles and a decrease in filler content is associated with an increase in surface roughness during surface treatment after a filling [6,15,16]. However, this correlation may be reversed after restoration wear. According to Johnsen et al., composites with a low volume of fillers and small filler sizes have the highest surface roughness difference before/after wear testing [17].

Surface quality is also greatly affected by the type of the finishing and polishing procedure applied [7]. A variety of finishing and polishing systems are currently available on the market, including “multiple-step” systems, consisting of at least two instruments, and “one-step” systems that require a single instrument [18,19]. Depending on the restorative material and the localization and size of the restoration, different finishing and polishing tools can be used, such as carbide burs, diamond burs, polyurethane-based finishing and polishing discs, abrasive-impregnated rubber cups, and polishing pastes [12,13,18,20,21]. Generally, this procedure includes the sequential use of instruments with a progressive decrease in abrasivity—e.g., diamond burs need to be followed by polishers, polishing disks, or polishing pastes [6]. It is clinically important to determine the finishing sequence that allows creation of the smoothest surface with the minimum time and fewest instruments [16].

Surface roughness, as an indicator of finishing and polishing efficacy, can be assessed by both qualitative and quantitative methods [19]. The roughness of the surface is most often established with the Ra value obtained using profilometry [22]. Ra is an amplitude parameter that characterizes the surface quality based on vertical deviations in the roughness profile from the centerline [18]. In the present study, a contact stylus profilometer was used for the evaluation. According to the literature, the clinically acceptable final roughness after polishing should be lower than the critical threshold of 0.2 micrometers, to prevent plaque accumulation [7,18,23].

Numerous studies have investigated the effect of different finishing and polishing procedures on the surface roughness of resin composites [24,25,26,27]. However, with the development of new composites and modern finishing and polishing systems, it is necessary to continuously update evaluations of the impact of different polishing procedures on surface quality. Moreover, the literature is scarce on the influence of the final treatment on the surface roughness of the composite/enamel (CEI) and composite/cementum (CCI) interfaces, which is also of great importance.

Therefore, the aim of this in vitro study was to investigate the effect of finishing and polishing sequences and composite type on the roughness of a composite surface, composite/enamel interface, and composite/cementum interface, as well as on the time required to achieve a visible gloss of the restoration.

The tested null hypotheses were that there would be no differences in surface roughness and polishing time: (1) among the composite resins treated using different polishing sequences; (2) among the three polishing sequences for each composite resin; and (3) surface roughness among the investigated surfaces, i.e., the composite surface, composite/enamel interface, and composite/cementum interface.

## 2. Materials and Methods

### 2.1. Specimen Preparation

Caries-free human third molars were selected for this study (*n* = 126). The use of extracted teeth for scientific research was approved by the Local Ethics Committee of Sechenov University (No. 0417, 17 April 2017). The teeth were extracted in the context of the treatment plan, and all patients gave written informed consent for the use of their extracted teeth for scientific research. After extraction, the teeth were scaled to remove organic and inorganic debris and stored in 0.1% thymol solution for 1 week for disinfection (ISO TS11405:2003 specifications) [28,29]. To prevent dehydration, the samples were kept in distilled water until they were used [28,30].

Class V cavities of a 4 mm width, 2 mm length, and 2 mm depth were prepared on the buccal surfaces with an 801–010 Medium diamond bur (Hager and Meisinger GmbH, Neuss, Germany) using a high-speed handpiece under air/water cooling (Synea Vision TK 94, W&H Dentalwerk Bürmoos GmbH, Austria). The 2 mm bevel was prepared at the occlusal margin of the cavity, and the gingival margin was located strictly at the cementoenamel junction.

Samples were randomly divided into 3 groups for further restoration using different A2-shade nanocomposite resins (Premise, *n* = 42 (Kerr, Scafati, Italy), Herculite Ultra, *n* = 42 (Kerr, Scafati, Italy), and Harmonize, *n* = 42 (Kerr, Scafati, Italy)). Table 1 shows the resin composites used in the present study and their composition.

Phosphoric acid (37%) was applied for 30 s to the enamel and 15 s to the dentine [31], and then rinsed with water for 60 s. The enamel and dentine were dried using the air from an air/water syringe. A three-step adhesive system OptiBond FL (Kerr, Scafati, Italy) was used: primer was applied on the dentine for 15 s followed by the application of a gentle air stream to evaporate the solvent. OptiBond FL adhesive was applied to the enamel and dentine for 15 s. The excess was removed with a microbrush, and the adhesive layer was light-cured. The polymerization procedure was carried out using a Demi Plus LED light-curing system (Kerr, Middleton, WI, USA). Demi Plus is a high-powered system (1100–1330 mW/cm^2^), and, in accordance with the manufacturer’s recommendations, the adhesive layers and enamel composite A2-shades were cured for 5 s. However, as a short curing time with high-intensity LEDs may influence the properties of the composite materials [32], a final 10 s cure was performed on each specimen. To ensure the accuracy of the cure, the output intensity of the curing light was constantly monitored using a Demetron L.E.D. Radiometer (Kerr, Middleton, WI, USA).

The restorative materials in each group were manipulated according to the manufacturer’s instructions and placed into the prepared cavity. The cured samples were then stored in distilled water at 37 °C for 24 h prior to final surface treatment.

**Figure 1:**
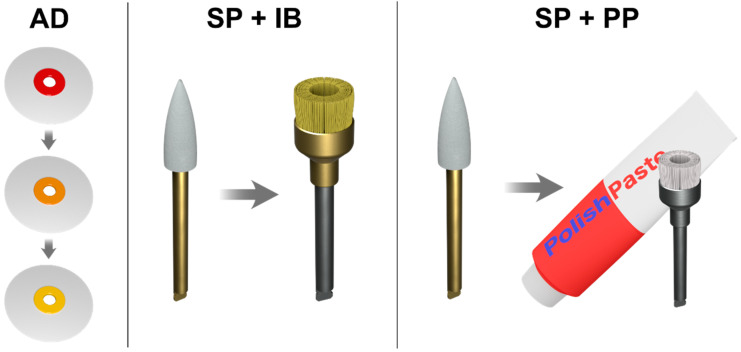
Finishing and polishing sequences used in the present study. AD—aluminum oxide abrasive discs; SP + IB—diamond-impregnated silicone polishers with aluminum oxide + brushes with fibers impregnated with silicon carbide abrasive particles; SP + PP—diamond-impregnated silicone polishers with aluminum oxide + polishing paste with aluminum oxide.

All the specimens were preliminary finished with an 858F–014 fine diamond bur (Hager & Meisinger GmbH, Neuss, Germany). Next, the specimens from each composite material received different surface treatments, according to the sequences recommended by the manufacturer (Figure 1).

In the present study, we used the following finishing and polishing sequences.

The AD sequence: The specimens were finished and polished sequentially with medium grit (40 μm), fine grit (20 μm), and extra-fine grit (10 μm) aluminum oxide abrasive discs (OptiDisc, Kerr, Bioggio, Switzerland).

The SP + IB sequence: The specimens were treated with “one-step” diamond-impregnated silicone polishers with aluminum oxide (Opti1Step, Kerr, Bioggio, Switzerland), followed by brushes with fibers impregnated with silicon carbide abrasive particles (OptiShine, Kerr, Bioggio, Switzerland).

The SP + PP sequence: The specimens were treated using “one-step” diamond-impregnated silicone polishers with aluminum oxide (Opti1Step, Kerr, Bioggio, Switzerland), followed by brushes and polishing paste with aluminum oxide (SuperPolish, Kerr, Bioggio, Switzerland).

The specimens were all finished and polished in the same direction parallel to the surface for all treated zones. To better simulate clinical settings, the finishing and polishing procedures were performed until the surface attained a visible gloss and not for a fixed period of time. The elapsed time was documented and analyzed. To avoid operator variability, all finishing and polishing procedures were performed by the same operator.

### 2.2. Surface Roughness Measurement

The specimens in each group were rinsed for 30 s. Then, they were dried with air/water syringe, and their surface roughness was evaluated in terms of the Ra value (μm) using a Surface Roughness Tester (Mitutoyo, Japan, Surftest SJ-410) with the stylus moving at a speed of 0.1 mm/s [33,34]. Ten tracings were performed, each 0.8 mm in length in three zones: composite surface, composite/enamel interface, and composite/cementum interface. The mean values were calculated.

### 2.3. Statistical Analysis

The normality of the distribution of the Ra-values and polishing times in the study groups were confirmed by a Shapiro–Wilk test. A Leven’s test showed the equality of the error variances. The data were analyzed using ANOVA, followed by a post hoc Tukey’s test at a 5% significance level, to compare the mean Ra values among the polishing systems, resin composites, and different types of interfaces. ANOVA followed by a post hoc Tukey’s test was also used to assess the influence of the finishing and polishing systems and composite type on the time required to achieve a visible surface gloss. The sample size was determined according to a previous study in which the roughness of the nanocomposites was assessed with the Ra value obtained using contact stylus profilometry [33]. The mean values used for calculation were 0.25 ± 0.11 and 0.405 (taken from groups with different polishing methods). The target sample size comprised 14 samples in each group (1−β = 80%, α = 0.05), taking into account multiple pair-wise comparisons.

## 3. Results

The ANOVA revealed a significant influence of the surface type on the final roughness (*p* < 0.001).

**Figure A1:**
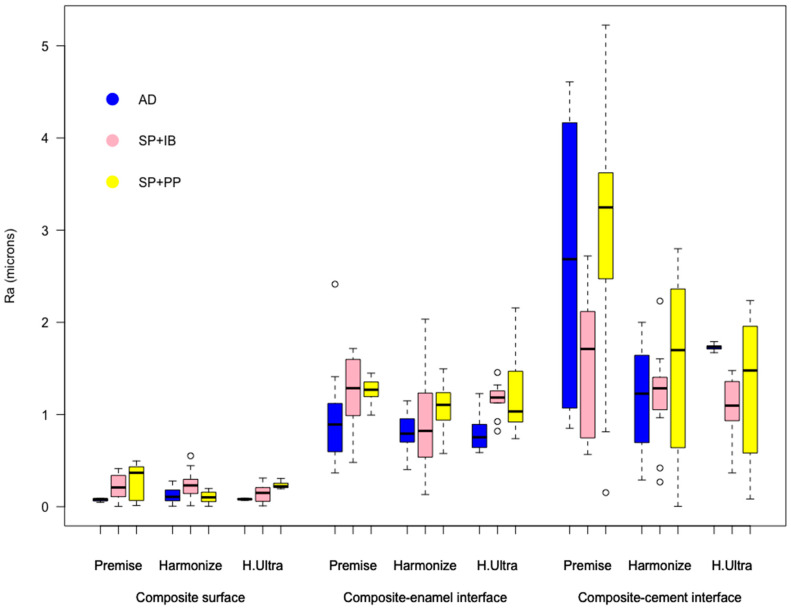
The roughness of the investigated surfaces depending on the composite used and the method of polishing.

The mean roughness values of the composite surfaces were significantly smaller than those of the composite/enamel interface and composite/cementum interface (*p* < 0.001) at 0.17 µm, 1.05 µm, and 1.69 µm, respectively. The difference between the composite/enamel and composite/cementum interfaces was not as important, but also significant (*p* < 0.001) (Figure A1).

**Figure 2:**
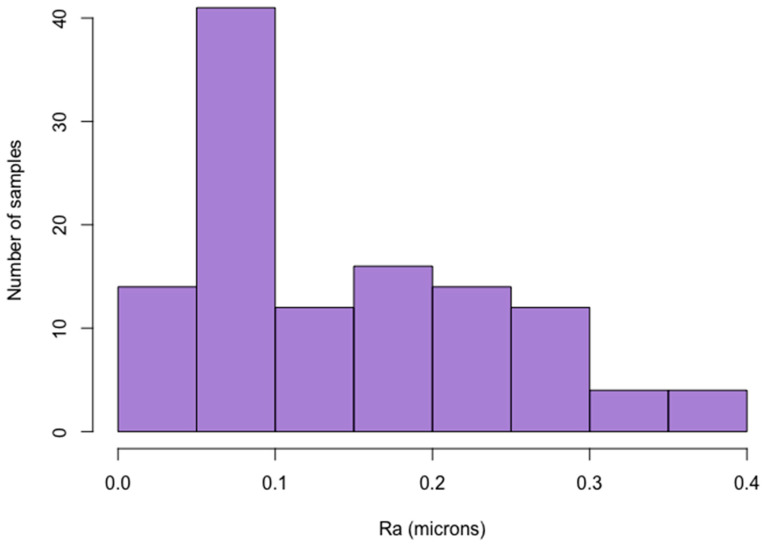
Histogram of the surface roughness of the composites polished until reaching a visible gloss.

The finishing and polishing procedures were performed until the surface attained a visible gloss, not for a fixed period of time. The mean Ra values of the different composite materials ranged between 0.08 µm and 0.29 µm. The highest Ra of a composite surface exhibiting a visible gloss was 0.38 µm (Figure 2). The influence of composite type on the restoration surface roughness was insignificant (*p* = 0.088), while the polishing method had a significant impact (*p* < 0.001) (Table 2). The composite surface polished with AD showed the lowest roughness (0.09 µm). This value was significantly smaller than the roughness values provided by SP + IB and SP + PP (*p* < 0.001).

The mean roughness of the composite/enamel interface ranged from 0.8 to 1.26 µm, and differed significantly among the composites (*p* = 0.01) and polishing methods (*p* < 0.001).The best surface quality of the composite/enamel interface (irrespective of the composite used) was detected after AD polishing, compared to the quality obtained using SP + IB (*p* = 0.001) and SP + PP (*p* < 0.001) polishing (Table 3). Harmonize produced Ra values significantly lower than those produced by Premise (*p* = 0.007), while the difference with Herculite Ultra was insignificant (*p* = 0.206).

The mean roughness of the composite/cementum interface ranged from 1.08 µm to 3 µm (Table 4). The differences were significant among the composites (*p* < 0.001) and polishing methods (*p* < 0.001). The highest values were detected for Premise, which demonstrated the poorest surface quality among the composite resins (*p* < 0.001). The SP + IB polishing sequence performed better than AD (*p* = 0.008) and SP + PP (*p* = 0.002).

Table 5 shows the mean time required to polish the composite surface to a visible gloss with different polishing methods. The polishing method, type of composite, and interaction of these factors had a significant impact on the time necessary to achieve a visible gloss on the composite surface (*p* < 0.001). The time needed to polish Premise and Herculite Ultra was almost 20% longer than the time needed to polish Harmonize (*p* < 0.001). Significant differences were also revealed among all polishing methods: SP + PP showed the shortest time, followed by SP + IB, whereas the AD sequence required significantly more time (*p* < 0.001).

## 4. Discussion

In the present study, we assessed the impact of finishing–polishing sequences and composite type on the roughness of the composite surface, composite/enamel interface, and composite/cementum interface, as well as on the time required to achieve a visible gloss.

The tested hypotheses were as follows: there would be no differences in surface roughness and polishing time (1) among the composite resins treated using different polishing sequences; (2) among the three polishing sequences for each composite resin; and (3) surface roughness among the investigated surfaces, i.e., the composite surface, composite/enamel interface, and composite/cementum interface.

Based on the results obtained, the first hypothesis was rejected for the composite/enamel and composite/cementum interfaces, as the mean roughness differed significantly among the composites. However, the hypothesis was accepted for composite surface, since there was no significant difference in surface roughness among the studied composite resins, while a significant difference in the time needed to polish these materials was observed. A significant difference in the time needed to polish these materials using different polishing sequences was also found. The second hypothesis was rejected, since the polishing method had a significant impact on the surface roughness. The third hypothesis was rejected as there were significant differences in the surface roughness values of the investigated surfaces: the composite surface, composite/enamel interface, and composite/cementum interface.

The final treatment of the composite resin, including the finishing and polishing procedures, plays an important role in the esthetic restoration, as it provides better optical properties and longevity. A rough surface can result in plaque accumulation, recurrent caries, periodontal diseases, and poor esthetics [7,12,15].

In order to assess the texture of dental restoratives, surface profilometers have been used for years in in vitro investigations [7,16]. In the present study, the surface quality of the specimens was also measured by using a contact stylus profilometer to obtain the numerical values of surface roughness.

In many studies, a resin composite, polymerized against a Mylar strip, was used as a control [6,16,33,35]. As has been previously reported in the literature, a polyester strip provides the smoothest surface [5,9,36]. However, in clinical settings, restorations routinely require final treatment for contouring, occlusal adjustment, and the removal of excess material [9,16,18]. Due to its high resin content, the layer cured in contact with the strip is more susceptible to wear, and should be removed [6,18,33,35]. Therefore, the quality of the surface achieved using the strip cannot be attained in dental practice, and a more clinically relevant roughness threshold is preferable to serve as the control. Numerous studies aimed to define the critical level of roughness; nonetheless, this value remains undetermined. According to Bollen et al., the threshold surface roughness for plaque retention is 0.2 μm [37]. Therefore, an Ra lower than 0.2 μm does not decrease bacterial adhesion [33,37]. These results are in agreement with those shown in a study conducted by Yuan et al. [38] The authors found that the area occupied by adherent bacteria was strongly correlated with the surface roughness for an Ra of 0.2–0.80 μm. Weitmen and Eams showed that plaque accumulation is similar for surface roughness values in a range between 0.7 and 1.4 μm [39]. In a recent study by Park et al., decreased adhesion of cariogenic streptococci was observed at Ra values of around 0.15 µm [40]. Other parameters influenced by surface roughness have been also described in the literature. Jones et al., stated that the mean roughness values between 0.25 and 0.50 µm could be detected by the tongue, thus leading to patient discomfort [41]. Regarding the esthetic properties, Chung et al., found that an Ra value lower than 1 μm provided an optically smooth surface [42].

In the present study, the finishing and polishing procedure was not limited by a specified time and was performed until a visible gloss was achieved. The composite materials in the different groups produced Ra values between 0.08 µm and 0.29 µm. For most specimens, this value was below or near the clinically acceptable 0.2 μm threshold. On the other hand, some of the specimens assessed by the operator as “acceptably polished” (having a visible gloss) exhibited a roughness two-times as high as the clinically acceptable values (up to 0.38 µm). Therefore, a visible gloss achievement cannot be used as a criterion of a sufficient polishing, and a more precise and objective assessment method should be developed for clinical use.

Similar findings were reported in a study by Endo et al., who compared the surface roughness of one nanofilled and three nanohybrid composite materials finished and polished with different systems. Although the difference in surface roughness among the composites was revealed, the final roughness values of most specimens were lower than the 0.2 µm threshold. The highest mean Ra value was 0.45 μm [18]. In a study by Ehrmann et al., all the tested nanotech-based composites showed Ra values below the critical level, with the highest being Ra = 0.17 μm [6].

The surface roughness of dental restoratives depends on both intrinsic and extrinsic factors. Intrinsic factors are associated with the composition of the composite materials (filler loading, differences in monomer proportions, and filler type, particle size, and shape) and the polymerization procedure [21,35]. Extrinsic factors include the finishing technique and applied finishing and polishing systems [12,35].

In the literature, there are controversial reports on the effects of composite type on surface roughness. Some papers suggest that even a considerable difference in particle size does not affect surface roughness. According to Gönülol and Yılmaz, resin composites with smaller filler sizes do not necessarily exhibit low surface roughness [43]. In the studies by Costa et al., and Kocaağaoğlu et al., hybrid and microhybrid composites were found to be similar to nanohybrid composites, in terms of their surface roughness [7,44]. In contrast, Avsar et al., showed that nanofilled composite resins allow the creation of significantly smoother surfaces than nanohybrid composite resins [33]. These contradictory results may be explained by the differences in the examined materials (in terms of filler size, filler type, and resin) and surface roughness measurement methods.

To obtain superior properties, manufacturers constantly develop new materials by optimizing the size and shape of the filler and monomer content. We studied three currently used nanohybrid composites launched by the same manufacturer between 2004 and 2016, to compare their polishability. The studied composites were as follows:

- Premise, pre-polymerized (30–50 µm) + irregular (0.4 µm) + nano size (20 nm) filler;
- Herculite Ultra, pre-polymerized (1 µm) + irregular (0.4 µm) + nano size (50 nm) filler;
- Harmonize, pre-polymerized + irregular (0.4 µm) + nanometric spherical conglomerate (30 nm) filler.

Although all these composite resins contain pre-polymerized, irregular, and nano-size fillers, the main trend is a decrease in the filler size. Moreover, some deviations in resin content should be taken into consideration. The results of the present study showed no significant differences in the surface roughness of the investigated resin composites, while slight deviations might be due to the filler quantity, type, and mean size. These findings could be explained by the relative similarities in the composition of the studied materials and manufacturing processes. The other possible explanation is that all materials examined in the present study were finished and polished for different periods of time, as the goal was to achieve visible gloss, which may be associated with the surface quality.

The time for finishing and polishing is a crucial parameter to be evaluated as it affects the surface roughness of the composite resin materials [34,45]. According to Heintze et al., finishing and polishing resin composite for 60 s results in a decrease of the surface roughness below the threshold level [45]. In the majority of studies evaluating the effect of finishing and polishing systems on composite surface roughness, the time that each instrument was used was strongly regulated [16,35,46]. However, the effectiveness of polishing tools depends on different factors, including individual anatomy, accessibility of the composite surface to finishing and polishing, the characteristics of the polishers, etc. [13]. As a result, it is virtually impossible to define the exact time necessary to achieve a predictable polishing effect in each individual case. Another parameter useful for assessing the surface quality chair-side is gloss [47]. In the present study, to better simulate clinical settings, the finishing and polishing procedures were performed until a visible gloss was achieved for each sample, and the required time was measured. Although the influence of composite type on restoration surface roughness was insignificant, the time required to achieve a visible gloss was material-dependent. Harmonize required the shortest time for polishing (2.03 min), compared with Premise (2.4 min) and Herculite Ultra (2.43 min), likely because of the lower content of pre-polymerized filler and smaller filler size of Harmonize. Regarding the finishing and polishing systems, AD required more time to obtain a smooth surface in all composites, followed by the SP + IB and O1P + SP sequences. The mean time values for these sequences, depending on the composite used, were 2.5–2.75 min, 1.95–2.53 min, and 1.52–2.15 min, respectively. This finding can be explained by the fact that in the AD group, three different tools were used consecutively, in contrast to other sequences, where only two instruments were used.

As mentioned above, apart from intrinsic factors, the surface roughness of resin composites depends on extrinsic factors, such as the finishing technique and the finishing and polishing tools applied. The surface micromorphology of dental restoratives after final treatment has been also shown to be influenced by the finishing and polishing system used [9,12,35]. The flexibility and shape of the polishing tool, as well as the size and hardness of the abrasive particles, play an important role during this procedure [5,9,16]. Since proper anatomical restoration requires contouring and the removal of excess material using diamond or carbide burs [10,12], we pretreated all the specimens with finishing diamond burs. Further treatment was carried out in accordance with the sequences chosen for each group. In the present study, we did not aim to compare the finishing and polishing products of different manufacturers. The purpose was to analyze the effect of different approaches on the final treatment procedure (e.g., aluminum oxide discs vs. polishers, abrasive-impregnated brushes vs. non-abrasive brushes combined with polishing paste, etc.). In order to achieve predictable results, polishing tools were chosen according to the composite manufacturer’s recommendations (i.e., from the same manufacturer).

A statistically significant difference was observed among the finishing and polishing systems in terms of the surface roughness, which was similar to other studies [7,18,19]. The lowest surface roughness of the resin composites was detected after AD polishing (*p* < 0.001). The mean Ra value was 0.09 µm, i.e., approximately two-times lower than in the other two groups. The highest mean Ra value for all composite materials tested in this study was 0.21 μm. This value was produced with SP + PP, although the difference between SP + IB and SP + PP was insignificant (*p* = 0.912).

These results are in agreement with those from other studies, which showed that aluminum oxide disks performed better than other polishing systems, due to their ability to cut the filler particles and matrix equally [33]. To provide regular abrasion, abrasive particles should be relatively harder than composite filler particles; otherwise, the softer composite resin matrix will be more susceptible to abrasion compared to inorganic filler. This irregular abrasion can result in an increase in surface roughness [16,21,33]. Regarding the size of the abrasive, the grit in the polishing tool should be smaller than the composite particle size, to achieve a smoother surface [48]. In many studies, flexible aluminum oxide discs were shown to cut the filler particles and resin matrix equally, thereby producing a smoother surface compared to silicone rubber polishers [7,33,35]. According to Nair et al., Soflex showed the smoothest surface (0.116 µm) and was significantly different from Enhance + Pogo [16]. Kocaağaoğlu et al., compared Bisco Finishing Discs with polishing wheel and Enhance and PoGo. The authors concluded that the discs presented smoother surfaces than Enhance, followed by PoGo. The median surface roughness was 0.28 µm [7]. In a study by Avsar et al., aluminum oxide discs performed better than other finishing and polishing techniques, particularly silicon rubber polishers [33]. The mean roughness was 0.26 µm. The differences in results may be explained by variations in the experimental design. Nevertheless, the efficacy of aluminum oxide discs depends on the anatomy and accessibility of the restoration surface undergoing the finishing and polishing procedures [13]. In the present study, the majority of the examined polishing systems demonstrated a mean surface roughness below the clinically acceptable threshold. Therefore, they may be considered as an alternative in clinical cases, where the use of aluminum oxide is limited or contraindicated.

Composite surface roughness is an important (but not the only) factor to be considered during final surface treatment. The quality of restoration margins is also essential for treatment outcome, since inadequate marginal integrity increases the risk of recurrent caries and periodontal disease [47]. In many studies, assessing the efficacy of polishing systems and the polishability of various composites, resin specimens were fabricated using metal or silicone molds [13,15,20]. This method does not allow the evaluation of marginal quality; therefore, in the present study, we prepared and restored class V cavities in extracted teeth. This approach enabled us to assess the effect of finishing and polishing on the surface roughness of the composite and restoration margins simultaneously. According to the data obtained, the surface of restoration was significantly smoother compared with the composite/enamel interface, and the composite/cementum interface in all composites and polishing systems. In contrast to the composite surface, the mean roughness of the composite/enamel interface and composite/cementum interface differed significantly between composites (*p* = 0.01 and *p* < 0.001 respectively), and exhibited a surface that was considerably rougher than the critical level for bacterial adhesion (0.8 µm–1.26 µm on the CEI, 1.08 µm–3.0 µm on the CCI). Harmonize produced Ra values for the composite/enamel interface that were significantly lower than those produced by Premise (*p* = 0.007), whereas the difference with Herculite Ultra was insignificant (*p* = 0.206). The other factor affecting the quality of the composite/enamel interface and composite/cementum interface was the sequence of the finishing and polishing procedure. The best surface quality of the composite/enamel interface for all composites was detected after AD polishing (0.86 µm), compared with SP + IB (*p* = 0.001) and SP + PP (*p* < 0.001) polishing, while the SP + IB polishing sequence performed better on the composite/cementum interface (1.28 µm) than AD (*p* = 0.008) and SP + PP (*p* = 0.002). This finding can be explained by the relative softness of the cementum, which is more susceptible to abrasion by aluminum oxide discs, compared to the composite material, leading to uneven wear. Furthermore, the use of discs in the cervical part of the crown may be limited, due to possible damage to the adjacent gingiva [49].

Other variables might also influence the final composite surface roughness, e.g., the operator [11,12,36], type of movements, and pressure applied to polish the samples [16,35,50]. In the present study, all the systems were applied using identical movements in the same direction parallel to the surface. Furthermore, to control these variables, the finishing and polishing procedures were carried out by a single operator. On the other hand, this fact could be considered a limitation of our study since the results obtained possibly depend on human factors. The limited number of composites and final treatment sequences tested is another limitation of this study. Finally, the surface roughness measurement was performed using a contact stylus profilometer, which examines the surface along certain paths and does not allow assessment of the entire area.

## 5. Conclusions

Within the limitations of this in vitro study, the following conclusions were drawn.

The roughness was significantly influenced by the surface type. The composite/cementum interface demonstrated the highest roughness, followed by the composite/enamel interface and the composite surface in all groups.

There was a statistically significant difference between the surface roughness and the finishing/polishing procedure adopted. Aluminum oxide abrasive discs performed better on the composite surface and composite/enamel interface, while diamond-impregnated silicone polishers with aluminum oxide + brushes with fibers impregnated with silicon carbide abrasive particles performed better on the composite/cementum interface.

The influence of composite type on the restoration surface roughness was insignificant, while the roughness of the composite/enamel and composite/cementum interfaces was material-dependent. The time required for visible gloss achievement depended on the type of composite resin.

A visible gloss achievement cannot be used as a criterion of a sufficient polishing, and a more precise and objective assessment method should be developed for clinical use.

## Figures and Tables

**Table 1:** Composition of the resin composite materials used in the present study.

Resin Composite	Manufacturer	Organic Resin	Filler Type	Filler Loading, %
Premise	Kerr, Scafati, Italy	BisGMA TEGDMA EBPADMA	Barium-aluminum-borosilicate glass (mean particle size 0.4 μm); fumed silica nanofiller (20 nm); prepolymerized filler (≈30–50 μm).	84 (by weight)
Herculite Ultra	Kerr, Scafati, Italy	BisGMA TEGDMA EBPADMA	Barium-aluminum-borosilicate glass (mean particle size 0.4 μm); fumed silica nanofiller (50 nm); prepolymerized filler (≈1 μm).	78 (by weight)
Harmonize	Kerr, Scafati, Italy	BisGMA TEGDM EBPADMA	Barium-aluminum-borosilicate glass (mean particle size 0.4 μm); aggregated zirconia/silica cluster filler (2–3 μm) comprised of 20 nm spherical fumed silica and 5 nm zirconia particles; prepolymerized filler.	81.5 (by weight)

BisGMA—Bisphenol A diglycidyl ether dimethacrylate; TEGDMA—Triethylene glycol dimethacrylate; EBPADMA—Ethoxylated bisphenol A dimethacrylate.

**Table 2:** The mean (M ± σ) and confidence interval (CI) of the composite surface roughness according to the Ra measurement (µm).

Composite/Polishing Method	Premise		Harmonize		Herculite Ultra		All Composites	
	M ± σ	CI 95%	M ± σ	CI 95%	M ± σ	CI 95%	M ± σ	CI 95%
AD	0.08 ± 0.02^a^	0.07–0.08	0.12 ± 0.08^ac^	0.08–0.15	0.08 ± 0.01^a^	0.08–0.09	0.09 ± 0.05^A^	0.07–0.12
SP + IB	0.22 ± 0.13^b^	0.15–0.29	0.23 ± 0.15^bc^	0.15–0.31	0.15 ± 0.09^ab^	0.10–0.20	0.2 ± 0.13^B^	0.13–0.27
SP + PP	0.29 ± 0.18^b^	0.19–0.38	0.10 ± 0.06^a^	0.07–0.14	0.23 ± 0.04^b^	0.22–0.25	0.21 ± 0.16^B^	0.14–0.28
Average	0.20 ± 0.16^A^	0.11–0.28	0.15 ± 0.12^A^	0.09–0.21	0.15 ± 0.08^A^	0.10–0.20	0.17 ± 0.12	0.10–0.23

Ra—surface roughness level; AD—aluminum oxide abrasive discs; SP + IB—diamond-impregnated silicone polishers with aluminum oxide **+** brushes with fibers impregnated with silicon carbide abrasive particles; SP + PP—diamond-impregnated silicone polishers with aluminum oxide **+** polishing paste with aluminum oxide; ^a,b,c,A,B^—homogenous subgroups.

**Table 3:** The mean (M ± σ) and confidence intervals (CI) of the roughness of the composite/enamel interface according to the Ra measurement (µm).

Composite/Polishing Method	Premise		Harmonize		Herculite Ultra		All Composites	
	M ± σ	CI 95%	M ± σ	CI 95%	M ± σ	CI 95%	M ± σ	CI 95%
AD	0.96 ± 0.50^a^	0.69–1.22	0.80 ± 0.21^a^	0.69–0.91	0.80 ± 0.19^a^	0.69–0.90	0.86 ± 0.34^A^	0.68–1.03
SP + IB	1.26 ± 0.36^a^	1.07–1.45	0.92 ± 0.51^a^	0.65–1.19	1.18 ± 0.16^a^	1.10–1.26	1.12 ± 0.39^B^	0.91–1.33
SP + PP	1.25 ± 0.14^a^	1.18–1.32	1.06 ± 0.28^a^	0.91–1.21	1.18 ± 0.39^a^	0.97–1.39	1.16 ± 0.30^B^	1.01–1.32
Average	1.16 ± 0.38^A^	0.95–1.36	0.93 ± 0.34^B^	0.74–1.12	1.05 ± 0.32^AB^	0.08–1.22	1.05 ± 0.34	0.85–1.24

Ra—surface roughness level; AD—aluminum oxide abrasive discs; SP + IB—diamond-impregnated silicone polishers with aluminum oxide + brushes with fibers impregnated with silicon carbide abrasive particles; SP + PP—diamond-impregnated silicone polishers with aluminum oxide + polishing paste with aluminum oxide; ^a,A,B^—homogenous subgroups.

**Table 4:** The mean (M ± σ) and confidence intervals (CI) of roughness of the composite/cementum interface, according to the Ra measurements (µm).

Composite/Polishing Method	Premise		Harmonize		Herculite Ultra		All Composites	
	M ± σ	CI 95%	M ± σ	CI 95%	M ± σ	CI 95%	M ± σ	CI 95%
AD	2.64 ± 1.42^a^	1.90–3.38	1.19 ± 0.53^b^	0.90–1.46	1.73 ± 0.03^ab^	1.71–1.75	1.85 ± 1.05^A^	1.30–2.40
SP + IB	1.55 ± 0.72^b^	1.17–1.93	1.21 ± 0.47^b^	0.96–1.46	1.08 ± 0.29^b^	0.93–1.24	1.28 ± 0.55^B^	0.99–1.57
SP + PP	3.00 ± 1.41^a^	2.26–3.74	1.57 ± 0.92^b^	1.09–2.05	1.27 ± 0.77^b^	0.86–1.68	1.95 ± 1.29^A^	1.27–2.62
Average	2.39 ± 1.35^A^	1.69–3.10	1.32 ± 0.68^B^	0.97–1.68	1.36 ± 0.54^B^	1.08–1.64	1.69 ± 1.05	1.15–2.24

Ra—surface roughness level; AD—aluminum oxide abrasive discs; SP + IB—diamond-impregnated silicone polishers with aluminum oxide + brushes with fibers impregnated with silicon carbide abrasive particles; SP + PP—diamond-impregnated silicone polishers with aluminum oxide + polishing paste with aluminum oxide; ^a,b,c,A,B^—homogenous subgroups.

**Table 5:** The mean (M ± σ) and confidence interval (CI) of time (seconds) required to polish the composite surface to a visible gloss with different polishing methods.

Composite/Polishing Method	Premise		Harmonize		Herculite Ultra		All Composites	
	M ± σ	CI 95%	M ± σ	CI 95%	M ± σ	CI 95%	M ± σ	CI 95%
AD	150 ± 14^a^	143–158	157 ± 05^a^	155–159	165 ± 20^a^	155–175	157 ± 15^A^	149–165
SP + IB	152 ± 14^ac^	145–159	117 ± 22^b^	105–129	145 ± 08^c^	141–149	138 ± 22^B^	127–149
SP + PP	129 ± 01^b^	128–130	91 ± 02^c^	89–92	128 ± 03^b^	126–130	116 ± 18^C^	107–125
Average	144 ± 15^A^	136–152	122 ± 30^B^	106–138	146 ± 20^A^	136–156	137 ± 25	124–150

AD—aluminum oxide abrasive discs; SP + IB—diamond-impregnated silicone polishers with aluminum oxide + brushes with fibers impregnated with silicon carbide abrasive particles; SP + PP—diamond-impregnated silicone polishers with aluminum oxide + polishing paste with aluminum oxide; ^a,b,c,A,B,C^—homogenous subgroups.

## Supplementary Materials

Supplementary File 1
