# Supplementary figures and images for: The Effect of Finishing and Polishing Sequences on The Surface Roughness of Three Different Nanocomposites and Composite/Enamel and Composite/Cementum Interfaces
Source: Nanomaterials (Basel). 2020 Jul 9;10(7):1339. doi: 10.3390/nano10071339 (PMC7407209; doi:10.3390/nano10071339)

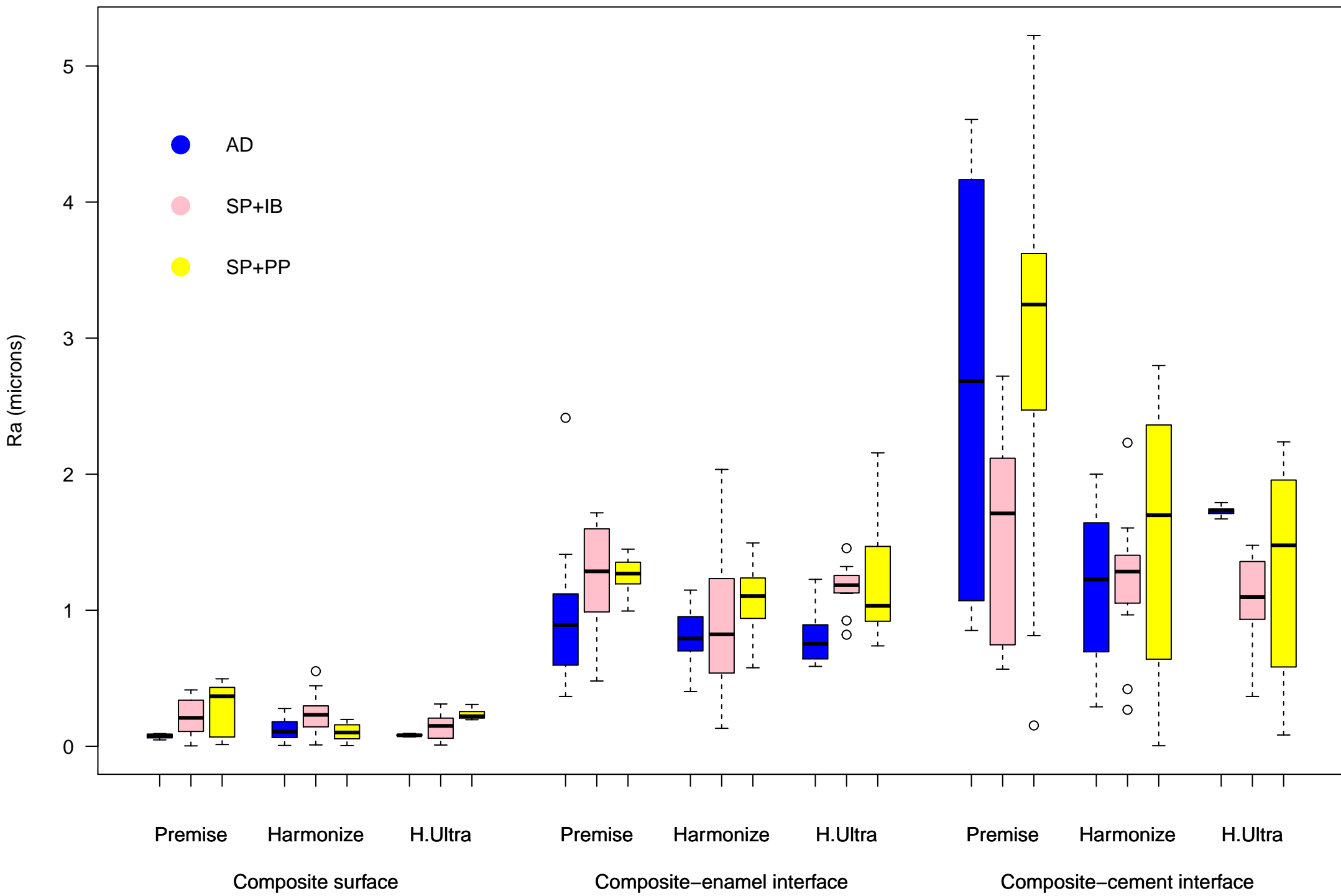

Supplement: Supplementary File 1 [file nanomaterials-10-01339-s001.pdf]
